# Sudomotor Testing of Diabetes Polyneuropathy

**DOI:** 10.3389/fneur.2018.00803

**Published:** 2018-09-26

**Authors:** Sarah-Maria Krieger, Manja Reimann, Rocco Haase, Elena Henkel, Markolf Hanefeld, Tjalf Ziemssen

**Affiliations:** ^1^Autonomic and Neuroendocrinological Laboratory Dresden, Center of Clinical Neuroscience, University Hospital Carl Gustav Carus, Dresden, Germany; ^2^GWT-TUD mbH, Study Center Professor Hanefeld, Dresden, Germany

**Keywords:** diabetes, polyneuropathy, screening tool, sudomotor function, Sudoscan

## Abstract

**Objective:** The performance of the Sudoscan technology for diagnosing diabetic polyneuropathy (DPN) was evaluated against the quantitative sudomotor axon reflex test (QSART). Furthermore, the association of Sudoscan with two clinical neuropathy scoring systems was evaluated.

**Methods:** Forty-seven patients with type 2 diabetes (20 without DPN, 27 with DPN) and 16 matched controls were examined for neuropathic symptoms and for the extent of sensory deficits. Sweat latency and volume by QSART and the skin electrochemical conductance (ESC) by Sudoscan were measured.

**Results:** The feet and hand ESC was significantly lower in patients with DPN as compared to controls. Patients with DPN had also lower hand ESC than patients without DPN. Sensitivity and specificity of feet and hand ESC for detecting DPN were 70/85% and 53/50% respectively. QSART could not differentiate between the three groups. ESC was inversely related to neuropathic symptoms and sensory impairment. ESC was significantly correlated with sensory impairment and pain.

**Conclusions:** Sudoscan shows a good performance in detecting subjects with DPN and it correlates well with clinical signs and symptoms of neuropathy.

**Significance:** This study provides evidence that Sudoscan has high potential to be used as screening tool for DPN and possibly also for small fiber neuropathy in diabetic patients.

**HIGHLIGHTS**

- The sudomotor function test Sudoscan shows a good performance to detect diabetes peripheral neuropathy.
- Sudoscan measures significantly correlate with clinical signs and symptoms of neuropathy.
- The Sudoscan technology may help to secure clinical diagnosis of small fiber neuropathy.

## Introduction

Diabetic neuropathy is the most common and disabling complication of diabetes mellitus accounting for the majority of non-traumatic amputations (1). An objective diagnosis of diabetic neuropathy is challenging due to its slow and gradual onset making the disease go undetected for many years. Its multifaceted and nonspecific clinical presentation with neuropathic pain being present in <1 third of individuals with polyneuropathy (2) additionally complicates its timely recognition. A proper diagnosis is often made when the disease has already caused irreversible damage to nerve fibers and significant function loss.

The most common type of diabetic peripheral neuropathy (DPN) is distal symmetric polyneuropathy which can be further subdivided into sensorimotor, motor, sensory, and autonomic neuropathy depending on the regions of the nervous system being affected (3, 4). Damage of small nerve fibers including unmyelinated C-fibers and myelinated Aδ-fibers can be observed at early stages of diabetic DPN. Small fiber neuropathy (SFN) has been demonstrated as early as in prediabetes (5, 6). Typical symptoms of SFN are sensory impairment, neuropathic pain, burning and tingling in feet that become worse at night and allodynia (7, 8). Clinically, those symptoms can be objectify in decreased pinprick test and temperature sensation, but neurological examination can also be normal (9). Since signs and symptoms, especially at the early stages, are very subtle SFN is generally difficult to diagnose. Currently, available diagnostic tools are limited to specifically detect damages to small nerve fibers. The quantification of intraepidermal nerve fiber density, although being minimally invasive (10, 9), is the preferred and most sensitive method for detecting SFN (9, 11–13). Since small C-fibers innervate sweat glands noninvasive, quantitative sudometry is increasingly applied to retrieve information about small nerve fiber function in patients with autonomic involvement (14, 15). The quantitative sudomotor axon reflex test (QSART) is regarded as sensitive and reproducible test of sudomotor function but its application is extremely limited due to high costs, special equipment needed and highly time-consuming nature (16).

The novel Sudoscan technology that is based on electrochemical conductance measurement provides a quick and cost-saving alternative of sweat function evaluation (17). A number of studies have shown that Sudoscan is able to detect DPN in diabetic patients (18–21) and that it correlates with QSART (20). In those studies, criteria for the definition of diabetic DPN differ widely, and patient results are usually compared to a set of data from the general population provided by the manufacturer. Furthermore, patient groups often consist of both patients with diabetes mellitus type 1 and type 2 although the two patient populations differ regarding the outcome of tight glycemic control on diabetic DPN (22). The current study therefore seeks to verify the performance of the Sudoscan technology in relation to the standard sudomotor test QSART to detect distal symmetric DPN, especially SFN, in a group of patients with diabetes mellitus type 2 in comparison to an age- and gender matched control group without diabetes. Furthermore, the Sudoscan outcome is compared to a clinical scoring system to proof its clinical relevance and its utility as screening method in every day clinical practice.

## Subjects and methods

A total of 63 Caucasian subjects (49% men, 51% women) aged 66 ± 5 (mean ± SD) years were enrolled between 2011 and 2013. All tests were performed on the same day. Solely the nerve conduction studies took place on another day. Patients were recruited from the Study Center Prof. Hanefeld, GWT-TUDmbH at the Technische Universität Dresden where they were in long term care and clinically very well characterized. Age and gender-matched control subjects were recruited by advertisements in medical practices and nursing homes. Control subjects were healthy despite of three subjects with high blood pressure and dyslipidemia. Patients were eligible when they were between 55 and 75 years old and had a clinical diagnosis of diabetes mellitus type 2 as diagnosed by the treating physician. Subjects with one of the following conditions were excluded: BMI ≥ 40 kg/m^2^, cardiac irregularity, allergy to nickel and alcohol abuse, DPN of other known causes, hypoglycemic or hyperglycemic current metabolic status and medication that induce DPN.

Patients were allocated to one of two groups depending on the presence or absence of DPN. DPN cases were defined according to the National Guidelines for the Diagnosis and Management of Diabetic Neuropathy in Adults by the German Association of the Scientific Medical Societies (AWMF) using clinical examination [neuropathy symptoms score (NSS) and neuropathy disability score (NDS)] and nerve conduction studies (2015). A subject was classified as having DPN when either of the tests—NSS and NDS or nerve conduction studies—was positive as explained in subsection Neuropathy Disability Score (NDS) and Neuropathy Symptoms Score (NSS) and Nerve Conduction Studies. Control subjects with a high suspicion of DPN were excluded. From initially 69 subjects, five candidates of the control group were ruled out because of pathologic findings either in nerve conduction studies or in clinical examination. One subject of the diabetes group with DPN was excluded because of chronic alcohol abuse.

The study conformed to the Declaration of Helsinki (2013) and was approved by the Ethics Committee of the Faculty of Medicine of the Technische Universität Dresden. All participants provided informed written consent.

### Neuropathy disability score (NDS) and neuropathy symptoms score (NSS)

A modified version of the clinical scoring system NSS and NDS was used to evaluate neuropathic symptoms and the severity of sensory deficits (23). The scoring system investigates the qualities of large and small nerve fibers (24). For the NSS, the presence of burning, numbness, paresthesia, weakness, cramps, and pain in feet or distal legs and its diagnostic relevance was weighted (range 0-3 points) as previously described (23). Exacerbations during day and night and improvement during movements were also considered. The maximum total score was ten points. Symptom classification was mild (3–4 points), moderate (5–6 points) or severe (7–10 points). Clinical examination for NDS included ankle tendon reflex, vibration, pin prick test and temperature sensation by cold tuning fork. All tests were performed on both sides. Normal reflex and sensory responses were scored zero (0), reduced or absent sensory responses were scored 1 for each side. A reduced or absent reflex was scored 1 and 2, respectively. The maximum total score was ten points. Minimum criteria for existence of distal symmetric DPN were: moderate clinical neurologic deficits (NDS 6–8 points) with or without symptoms, or mild clinical neurologic deficits (NDS 3–5 points) with at least moderate symptoms (NSS 5–6 points or higher). Hence, symptoms without sensory deficits in clinical examination were not sufficient for diagnosing a distal symmetric DPN.

### Nerve conduction studies

Nerve conduction studies were used to evaluate nerve function and to determine the presence of DPN in this study. Nerve conduction studies were performed via surface electrode patches attached to the skin by qualified staff. Analysis was performed by a professional neurologist educated in electro physiologic examinations. Voltcraft infrared thermometer was used in order to prevent influence of skin temperature on measurements. Assessment was based on nerve conduction velocity and amplitude (peak-to-peak). Abnormal values of both were used for the diagnosis of DPN. The measurements of the motoric part of the peroneal nerve (between fibular head and ankle), of the motoric part of the median nerve (between elbow and wrist), of the sensory part of the median nerve (between wrist and index), and of sural nerve (lower leg) were evaluated for diagnosing DPN. Standardized protocol and laboratory-owned reference values correspond to Bischoff (25).

### Assessment of sudomotor function

All sudomotor function tests were performed in a quiet and temperature-controlled autonomic laboratory with ambient air humidity (room temperature: 22–23°C, room humidity 40–60%). Subjects were in upright position during the Sudoscan testing and in recumbent position during the QSART.

#### Sudoscan test

The electrochemical principle has been previously described and involves the induction of a chloride based electrochemical current after activation of sweat glands by a low-voltage current (< 4 V) (26). Due to the isolator function of corneal stratum of epidermis, it is expected that the measured net current corresponds to the local sweat response. The Electrochemical Skin Conductance [ESC in microSiemens (μS)] that represents the chloride ion current is calculated by the ratio of extrapolated current and constant direct current. ESC was measured at both hands and feet by placing the palms and soles on stainless steel electrodes for 2 min. The measurement was repeated twice and the average ESC calculated. Low ESC indicates a high risk of a somatosensory neuropathy (27–30). Based on a previous study (30), an ESC of> 70 μS (feet)/> 60 μS (hands) is considered to indicate normal sudomotor function while an ESC of 50–70 μS (feet)/40–60 μS (hands) and of < 50 μS (feet)/ < 40 μS (hands) is suggestive of moderate and severe sudomotor dysfunction respectively. Current studies support these normative values (31) 32QSART, which evaluates the function of postganglionic sympathetic cholinergic neurons, was measured by Q-Sweat System (WR Medical Electronics Co., Stillwater, MN, United States). A detailed description of the Q-Sweat system can be obtained from the manufacturer. The principle of QSART has been described in detail elsewhere (32). Shortly, sweat glands are activated by acetylcholine iontophoresis and the sweat response is measured as increase in humidity through a hygrometer. Multi-compartment capsules are attached to the skin of the medial forearm (75% of the distance from the ulnar epicondyle to the pisiforme bone) and medial distal lower leg (5 cm proximal to the medial malleolus) by silicon straps. The skin was cleaned with acetone, isopropyl alcohol, and water. The outer chamber is filled with 10% acetylcholine and the inner compartment detects moisture from sweat response. Both chambers are separated by an air filled middle compartment. Once a stable signal had been reached the following standard protocol was applied where humidity was continuously recorded: 5 min baseline, 5 min acetylcholine iontophoresis at a current of 2 mA and 5 min post stimulation (16). A pathologic finding was defined as a prolonged sweat response latency (normal reference 1–2 min), a reduced or absent sweat response, or an elevated sweat volume (14, 33). The sweat volume was presented as area under the curve (AUC). Since normative values vary largely across studies (14, 15, 33) the values of the matched control group was regarded as reference.

### Statistical analysis

Statistical analysis was performed with SPSS software package version 23.0 for Windows (IBM Corp, Armonk, NY, United States). Descriptive data are presented as mean and standard deviation (SD). Severely skewed data were logarithmically transformed if deemed appropriate. When the normal distribution was approximated by transformation the log transformed variable was used in the analysis. Study parameters were compared between the three groups by one-way ANOVA with Bonferroni Correction and χ2 Test. The ability of Sudoscan and QSART to correctly classify individuals with and without DPN was evaluated by plotting receiver operating characteristic-(ROC) curves from which sensitivity and specificity was derived. The presence/absence of DPN was coded by 1/0.The best cut-off value for classification was determined by Youden's index (Sensitivity+Specificity-1) after adjustment to increase sensitivity. The direction and strength of the relationship between the methods was evaluated by Pearson product-moment correlation. The association of sudomotor function with age, gender, HbA1c, and plasma glucose was also determined. Statistical significance was accepted at *p* < 0.05.

## Results

### Clinical features of study groups

Gender was evenly distributed across control subjects and patients (Table 1). Patients with DPN were significantly older and had a higher BMI than control subjects. Serum triglycerides, plasma glucose (4 h-postmeal plasma glucose) and HbA1c were higher and HDL cholesterol levels were lower in patients with DPN as compared to controls. Patients without DPN and controls differed regarding HDL cholesterol and HbA1c. Diabetes duration and metabolic parameters were comparable between patients with and without DPN.

**Table 1:** Clinical characteristics of the study population.

**Parameters**	**Controls**	**Diabetics**	**DPN**	***P*-value**
	***N*** = **16**	***N*** = **20**	***N*** = **27**	
Sex (M/F)	9/7	8/12	14/13	0.585
Neuropathy (N/J)	16/0	20/0	0/27	<0.001
Age (Years)	64 (5.1)^A^	66 (5.8)^AB^	69 (4.8)^B^	0.006
BMI (kg/m^2^)	24.9 (2.6)^A^	27.4 (3.5)^AB^	30.1 (4.5)^B^	<0.001
Diabetes duration (Years)	–	13.3 (10.0)	15.4 (9.7)	0.465
Creatinine (μmol/l)	76.6 (15.8)	85.5 (16.0)	90.0 (21.9)	0.086
Triglyceride (mmol/l)^a^	1.7 (1.2)^A^	2.2 (1.3)^AB^	2.6 (1.3)^B^	0.012
HDL Cholesterol (mmol/l)	1.9 (0.4)^A^	1.4 (0.4)^B^	1.3 (0.3)^B^	<0.001
LDL Cholesterol (mmol/l)	3.6 (0.6)	3.0 (0.9)	3.2 (1.1)	0.162
Plasma glucose (mmol/l)	5.1 (0.9)^A^	6.2 (1.4)^AB^	6.5 (2.1)^B^	0.006
HbA1c (%)	5.4 (0.3)^A^	6.4 (0.5)^B^	6.8 (0.9)^B^	<0.001
NSS total score	1.4 (2.7)	4.0 (3.7)	6.8 (2.0)	<0.001
NDS total score	1.2 (1.2)	1.65 (1.4)	6.22 (2.5)	<0.001

*Data are mean (SD). Unequal superscript letters indicate significant group differences*.

a *log-transformed variable.HbA1c, glycated hemoglobin A1c; NDS, neuropathy deficit score; NSS, neuropathy symptoms score; DPN, diabetic peripheral neuropathy*.

### Sudomotor function in diabetic patients with and without DPN

The average ESC of feet was significantly smaller in patients with DPN than controls (Table 2). A significant difference was neither observed between patient groups nor between controls and patients without DPN. The average ESC of hands was significantly lower in patients with DPN as compared to patients without DPN and controls.

**Table 2:** Sudomotor function of study subjects.

	**Controls**	**Diabetics**	**DPN**	***p*-value**
	***N*** = **16**	***N*** = **20**	***N*** = **27**	
ESC feet (μS)	83.0 (16.3)^A^	76.3 (15.9)^AB^	64.4 (16.5)^B^	0.017
ESC hands (μS)	71.3 (18.0)	70.4 (17.6)	58.2 (18.2)	0.037
Sweat latency, distal leg (s)^a^	87.2 (49.4)	96.4 (49.5)	90.5 (49.7)	0.909
Sweat volume, distal leg(μl)^a^	0.9 (0.6)	0.7 (0.6)	0.6 (0.6)	0.107
Sweat latency, distal forearm (s)	80.3 (41.1)	89.1 (40.2)	84.9 (42.3)	0.807
Sweat volume, distal forearm (μl)^a^	1.0 (1.4)	1.2 (1.4)	1.3 (1.4)	0.944

*Data are age- and gender adjusted mean (SD). Unequal superscript letters indicate significant group differences*.

a log-transformed variable. ESC, electrochemical skin conductance, DPN, diabetic peripheral neuropathy

Controls and patients did not differ regarding sweat volume and response latency of QSART (Table 2). There was a trend toward a lower sweat volume and prolonged response latency in patients as compared to controls.

### Performance of sudomotor tests to diagnose DPN

**Figure 1:**
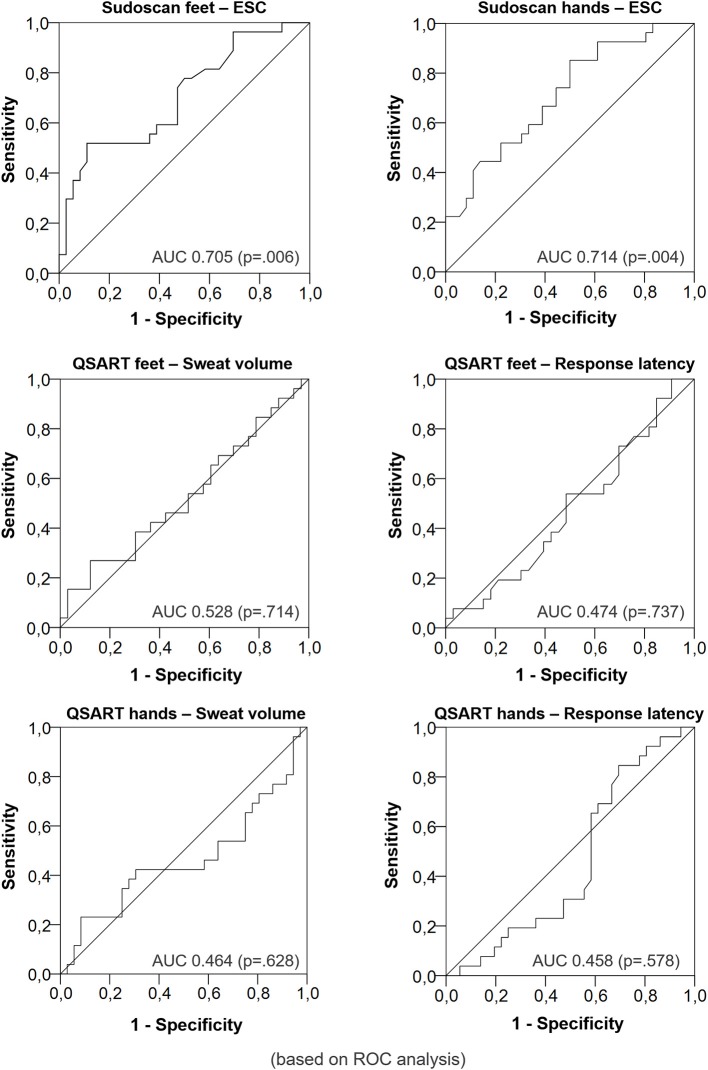
Diagnostic performance of Electrochemical Skin Conductance vs. sweat volume and response latency for DPN (Receiver operating characteristic curves).

When choosing a feet ESC of ≤ 80.00 μS (optimal Youden index), sensitivity was 70% and specificity was 53%. In general, a smaller ESC was associated to the presence of neuropathy. The respective area under the ROC curve was 0.705 (*p* = 0.006). The sensitivity was 85% and the specificity was 50% with a hand ESC cut-off of ≤ 75.00 μS (optimal Youden index). The area under the ROC curve was 0.714 (*p* = 0.004, Figure 1).

**Figure 2:**
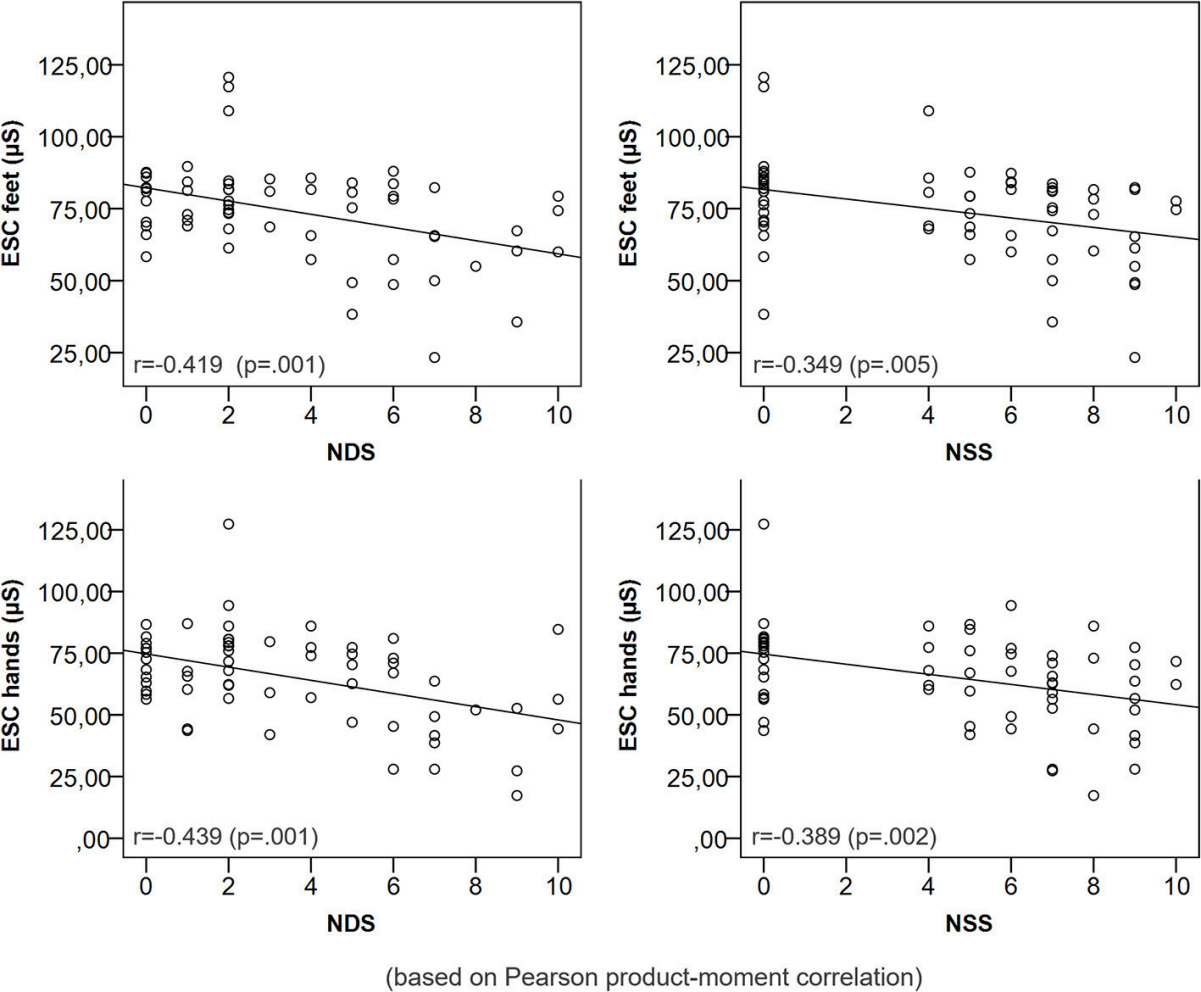
Correlation of ESC with NDS/NSS.

The sweat volume and the sweat latency were not able to distinguish between the groups. The area under the ROC curve was 0.528 (*p* = 0.714) for the sweat volume and 0.474 (*p* = 0.737) for the response latency measured at the legs respectively. The area under the ROC curve was 0.464 (*p* = 0.628) for the sweat volume and 0.458 (*p* = 0.578, Figure 2) for the response latency measured at the forearm respectively.

### Correlation of ESC with QSART sweat response and clinical parameters

ESC was not or only weakly related to age in all study groups (ESC feet *r* = −0.199, *p* = 0.118; ESC hands *r* = −0.276, *p* = 0.029). ESC was unrelated to gender (*p* > 0.05). A significant negative correlation was demonstrated between ESC and HbA1c (ESC feet *r* = −0.369, *p* = 0.003; ESC hands *r* = −0.412, *p* = 0.001) but not for plasma glucose (*p* > 0.05).

The sweat volume at the distal legs and feet ESC were significantly positively correlated (*r* = 0.265, *p* = 0.043). Other parameters of QSART and ESC were not correlated (*p* > 0.05). A lower ESC of feet and hands was correlated with a higher NDS and NSS (Figure 2). ESC was significantly related to a pathologic pin prick test and the presence of neurological symptoms (Table 3).

**Table 3:** Relationship of Sudoscan measures with clinical scoring systems.

**Clinical parameters**	**ESC feet**		**ESC hands**	
	**r**	***p*-value**	**r**	***p*-value**
NDS total score	−0.419	0.001	−0.439	0.001
NSS total score	−0.349	0.005	−0.389	0.002
Temperature sensation	−0.214	0.092	−0.266	0.035
Pinprick test	−0.433	0.001	−0.402	0.001
Pain perception	−0.362	0.004	−0.432	<0.001
Burning sensation	−0.270	0.032	−0.351	0.005

*r, Pearson product-moment correlation coefficient; ESC, electrochemical skin conductance; NDS, neuropathy deficit score; NSS, neuropathy symptoms score*.

### Inter-individual variation of sudomotor measures

The between-subject coefficient of variation (inter-CV) of hands and feet ESC were 28 and 22%, respectively. The inter-CV of sweat latency and sweat volume at the distal forearm amounted to 48 and 120%, respectively. The sweat latency of the distal legs showed an inter-CV of 49% and the sweat volume of the distal legs varied by 79% between subjects.

## Discussion

The present study evaluated the performance of the novel noninvasive sudomotor function test Sudoscan as screening tool for DPN in comparison to the validated QSART. Furthermore, the relationship of Sudoscan measures with clinical predictors of DPN was investigated. The study demonstrated a good sensitivity and a moderate specificity for Sudoscan to detect DPN in well-characterized patients with diabetes mellitus with a slightly better sensitivity for hands than feet ESC. A lower ESC was associated with a higher probability of having distal symmetric DPN. ESC was also significantly associated to clinical predictors of neuropathy and clinical neuropathy scoring systems.

Previous studies have provided similar sensitivity of ESC to detect DPN in diabetes mellitus. However, the specificity was consistently higher than in our study (18–20, 34). It is critically important for a screening tool to have a high success rate of detecting positives. The sensitivity was therefore increased at the expense of specificity what may explain the lower specificity in the present study. The cut-offs for DPN detection were also higher in our study group than previously reported (19). We carefully classified DPN based on the findings of the clinical evaluation and nerve conduction studies which is considered the gold standard according to the American Academy of Neurology (35). In contrast to the other studies, our patients with DPN exhibited only mild symptoms of DPN reflecting an early disease stage which may justify a higher cut-off for the detection of DPN at early stages.

QSART is considered as the standard method of noninvasive sudomotor testing with sensitivity ranging between 77 and 80% for the detection of small fiber neuropathy (11, 36, 13). Unlike previous studies, the ability of QSART to detect DPN in diabetic subjects was very limited and lower in comparison to ESC in our study. The likelihood to screen positive for DPN in those affected was 50% or less. Furthermore, the agreement between sudomotor function measured by Sudoscan and QSART at the lower limb was weak if any. Smith et al. (20) also detected only a moderate correlation of sweat volume of lower limb and ESC (20). A recently published study could neither detect correlation between ESC and QSART at the foot nor with QSART and intraepidermal nerve fiber density. Whereas ESC and intraepidermal nerve fiber density correlate significantly (37, 38). One explanation for different findings may be that our patients with DPN were not exclusively screened for SFN but they rather showed a mixed pattern of DPN with involvement of large and small nerve fibers. As QSART targets exclusively the function of small postganglionic sympathetic fibers, it may be limited in detecting a mixed pattern of neuropathy. This assumption is refuted by the study of Low et al. (39) who examined 125 patients with isolated SFN and with a mixed pattern of neuropathy. In this study, the sensitivity of QSART to detect DPN was 77% and increased to 93% when it was combined with Thermoregulatory Sweat Testing. More importantly, the results indicated that the site of measurement is critical with proximal feet yielding the best sensitivity. This may reflect the length-dependent dissemination of DPN beginning distally in the feet. In our study, the most distally electrodes were placed above the ankle a site that may be less sensitive in patients at early stages of DPN. The interaction between site of measurement and duration of disease may also explain the large inter-individual fluctuation of QSART measurements in our study which has also been previously described by others (40). The large inter-individual variability of sweat volume and latency seems to be most relevant to explain the lack of discriminative power in our study. This assumption is supported by two recent investigations (41, 42) showing a large inherent variability in sweat volume measurements during repeated testing and a moderate test-retest reliability. More importantly, they also presented large standard deviations and showed that the variability of repeated measurements can be even larger than the value itself. Considering the minimization of external influences by high standardization of measurements, internal (physiologic) factors may be primarily responsible for a high individual variability of measurements. Especially the sweat gland output and the number of activated fibers within the reflex circuit have been shown to highly vary between individuals (43). ESC has been found to vary less between subjects which might be owing to the fact that unlike QSART it measures general sudomotor function (33) and is less prone to error due to the complex measurement procedure (16). Recent clinical signs of SFN are very subtle and have been found as early as in subclinical disease (impaired glucose tolerance) (5, 6). Thus, an affection of small nerve fibers in the diabetes group classified as not having DPN cannot be entirely excluded.

The strong association of ESC measures with clinical signs and symptoms (i.e. NDS and NSS) highlights the fact that ESC correlates with both small and large fiber function and therefore has high potential as screening method for DPN. Thus, a combination of neuropathic symptoms and signs with a low ESC measurement may enable a more reliable diagnosis than one of them alone. There is a large number of clinical scoring systems for clinical diagnosis of DPN. The NDS and NSS used in the current study are validated and recommended by the German Association of the Scientific Medical Societies (2015). Casellini et al. tested different clinical scoring systems in 83 diabetic patients and 210 controls (18). Each scoring system was significantly correlated to ESC. Another study also found a significant association between feet ESC and clinical symptoms (21). Altogether, the data indicate that Sudoscan is useful to objectify and reinforce the clinical diagnosis of DPN.

The significant correlation of a low ESC with impaired sensation may also emphasize a potential use of Sudoscan for the diagnosis of SFN. This is supported by Casellini and colleagues showing that an impaired temperature sensation in patients with SFN is related to a low feet ESC (18). From the evaluated sensory modalities, temperature sensation showed the weakest correlation in our study. Lefaucheur et al. demonstrated that the sensitivity of ESC measurements is higher than sensitivity of temperature sensation for detection of SFN (44). The loss of temperature sensation may be less well recognized by the patient than the onset of neuropathic pain which impacts far more on the quality of life and the psychological well-being (45).

A notable strength of our study is the well-matched and well-characterized study group and a DPN classification according to national (2015) and international guidelines (35). The small number of subjects can be considered a limiting factor of this study. The groups were gender balanced which is important since sweat volumes significantly differ between males and females (39). Although the control subjects were slightly younger than the patients, the influence of age was found negligible as reflected by a weak or non-existent correlation with ESC in this and a previous study (30). Additionally performed age- and gender-adjusted analyses did not yield different results than unadjusted analyses rendering any influence by these factors insignificant referring to the age category of this study. Furthermore, patients with and without DPN were similar in major characteristics which ensured a high degree of comparability. The majority of patients with DPN in our study experienced mild symptoms and signs of neuropathy which was intended since a valid screening tool should be able to detect subclinical disease. This study selectively evaluated patients with diabetes mellitus since this patient group is at high risk of neuropathy due to hyperglycemia (1). Thus, it is unclear if the results can be generalized to patients with a different underlying pathology. Although we applied standard protocols of sudomotor function testing, we did not control the local skin temperature which is known to influence sweat response (46). At last, as already stated, sensitivity and specificity have to be adapted in future studies to optimize the screening capabilities of this tests as in our study the sensitivity was therefore increased at the expense of specificity what may explain the lower specificity in the present study.

## Conclusions and outlook

Our findings indicate that Sudoscan has great potential as a screening tool for distal symmetric DPN and SFN in patients with diabetes mellitus. ESC proved to be more sensitive to detect DPN than QSART and showed a lower inter-individual variability. The present study also highlights the utility of Sudoscan to objectify clinical symptoms and to secure a clinical diagnosis of DPN as strong relationships between ESC and somatic measures were shown. In contrast ESC was not or just weekly correlated with QSART. Therefore, further investigations are needed to assess if ESC and QSART possibly measure different aspects of DPN. Moreover, future studies in a larger group of subjects with different risk factors for neuropathy are necessary to confirm the present results. These studies should additionally apply intraepidermal nerve fiber density as the only direct measurement of morphological changes in small nerve fibers. For future investigations, the influence of weight and pressure, respectively, on ESC seems worth to be considered. So far, the measurements of Sudoscan were taken in an upright position. Further investigations are needed in order to exclude differences in pressure as reason for the difference in ESC of hands and feet.

## Author contributions

S-MK is responsible for data collection, analysis and interpretation of data and drafting of the article. TZ is the responsible study clinician and contributed to the study design, data analysis and interpretation. MR is responsible for the study design and general supervision. TZ and MR were involved in the discussion and comprehensibly revised/edited the manuscript before submission. RH contributed the data analysis and interpretation. EH and MH gave intellectual input during the study and contributed to the interpretation of the results. All authors gave final approval of the current version to be published. SK is the guarantor of this work and, as such, had full access to all the data in the study and takes responsibility for the integrity of the data and the accuracy of the data analysis.

### Conflict of interest statement

The authors declare that the research was conducted in the absence of any commercial or financial relationships that could be construed as a potential conflict of interest.

## Abbreviations

CV: coefficient of variation
DPN: diabetic peripheral neuropathy
ESC: electrochemical skin conductance
NSS: neuropathy symptoms score
NDS: neuropathy disability score
QSART: quantitative sudomotor axon reflex test
ROC: receiver operating characteristic curve
SFN: small fiber neuropathy.
